# Intracoronary Delivery of Mitochondria to the Ischemic Heart for Cardioprotection

**DOI:** 10.1371/journal.pone.0160889

**Published:** 2016-08-08

**Authors:** Douglas B. Cowan, Rouan Yao, Vamsidhar Akurathi, Erin R. Snay, Jerusha K. Thedsanamoorthy, David Zurakowski, Maria Ericsson, Ingeborg Friehs, Yaotang Wu, Sidney Levitsky, Pedro J. del Nido, Alan B. Packard, James D. McCully

**Affiliations:** 1 Department of Anesthesiology, Perioperative and Pain Medicine, Boston Children's Hospital and Harvard Medical School, Boston, MA, United States of America; 2 Department of Radiology, Boston Children's Hospital and Harvard Medical School, Boston, MA, United States of America; 3 Department of Cardiac Surgery, Boston Children's Hospital and Harvard Medical School, Boston, MA, United States of America; 4 Department of Cell Biology, Harvard Medical School, Boston, MA, United States of America; 5 Department of Cardiothoracic Surgery, Beth Israel Deaconess Medical Center and Harvard Medical School, Boston, MA, United States of America; University of Miami School of Medicine, UNITED STATES

## Abstract

We have previously shown that transplantation of autologously derived, respiration-competent mitochondria by direct injection into the heart following transient ischemia and reperfusion enhances cell viability and contractile function. To increase the therapeutic potential of this approach, we investigated whether exogenous mitochondria can be effectively delivered through the coronary vasculature to protect the ischemic myocardium and studied the fate of these transplanted organelles in the heart. Langendorff-perfused rabbit hearts were subjected to 30 minutes of ischemia and then reperfused for 10 minutes. Mitochondria were labeled with ^18^F-rhodamine 6G and iron oxide nanoparticles. The labeled mitochondria were either directly injected into the ischemic region or delivered by vascular perfusion through the coronary arteries at the onset of reperfusion. These hearts were used for positron emission tomography, microcomputed tomography, and magnetic resonance imaging with subsequent microscopic analyses of tissue sections to confirm the uptake and distribution of exogenous mitochondria. Injected mitochondria were localized near the site of delivery; while, vascular perfusion of mitochondria resulted in rapid and extensive dispersal throughout the heart. Both injected and perfused mitochondria were observed in interstitial spaces and were associated with blood vessels and cardiomyocytes. To determine the efficacy of vascular perfusion of mitochondria, an additional group of rabbit hearts were subjected to 30 minutes of regional ischemia and reperfused for 120 minutes. Immediately following regional ischemia, the hearts received unlabeled, autologous mitochondria delivered through the coronary arteries. Autologous mitochondria perfused through the coronary vasculature significantly decreased infarct size and significantly enhanced post-ischemic myocardial function. In conclusion, the delivery of mitochondria through the coronary arteries resulted in their rapid integration and widespread distribution throughout the heart and provided cardioprotection from ischemia-reperfusion injury.

## Introduction

Ischemia and reperfusion of the heart significantly alters mitochondrial structure and function, which impairs oxidative phosphorylation and decreases high energy phosphate synthesis [1–11]. These events are initiated during ischemia and extend throughout reperfusion to severely compromise cardiac post-ischemic functional recovery and cell viability. We hypothesized that transplantation of autologous mitochondria isolated from a non-ischemic area in the patient’s own body to the myocardial ischemic zone during early reperfusion would augment or replace the function of mitochondria damaged during ischemia and would allow for enhanced post-ischemic functional recovery and preservation of cell viability [12, 13].

In earlier studies, we have validated this approach using the Langendorff-perfused rabbit heart model and the *in situ* blood-perfused rabbit heart [12, 13]. Endogenous cardiac mitochondria were supplemented with respiration-competent mitochondria isolated from a remote, autologous, non-ischemic tissue such as the liver or skeletal muscle [11]. By injecting exogenous mitochondria into the ischemic region at the onset of reperfusion, we established these hearts had increased tissue adenosine triphosphate (ATP) content and showed enhanced cardioprotection through reduction in infarct size, decreased cell loss, and improved post-ischemic contractile function [12, 13]. We went on to demonstrate that mitochondrial injection did not provoke an immune response and were not arrhythmic [13]. To enhance the clinical applicability of this promising new treatment strategy, we have begun to investigate alternative methods to deliver exogenous mitochondria to the injured heart.

In this study, we investigated whether mitochondria can be effectively transplanted to the heart through the coronary vasculature. To allow for visualization of uptake and distribution, mitochondria were labeled with ^18^F-rhodamine 6G (^18^F-R6G) and 30 nm magnetic iron oxide particles [14–18]. These dual-labeled mitochondria were either injected into the ischemic region of the myocardium or perfused through the coronary arteries using an established isolated rabbit heart model. Following reperfusion, both globally and regionally ischemic hearts containing transplanted mitochondria were examined using positron emission tomography (PET), microcomputed tomography (μCT), and magnetic resonance imaging (MRI). Tissue sections from these hearts were used for fluorescence and brightfield microscopy to confirm the location and quantity of transplanted organelles. Additional experiments using unlabeled, autologous mitochondria were performed to investigate the cardioprotection afforded by vascular delivery of mitochondria through the coronary arteries in regionally ischemic rabbit hearts. Our results demonstrate that mitochondria can be delivered to the myocardium by vascular perfusion and this significantly decreases myocardial infarct size and enhances post-ischemic functional recovery.

## Materials and Methods

### Animal Subjects

This study was carried out in strict accordance with the recommendations in the Guide for the Care and Use of Laboratory Animals by the National Institutes of Health. All animal procedures were approved by the Institutional Animal Care and Use Committee at Boston Children’s Hospital. Female New Zealand White rabbits (Covance, Dedham, MA) weighing 4–6 kg were sedated using ketamine (10 mg/kg) and acepromazine (0.5 mg/kg) with heparin (500 U) administered through the marginal ear vein. Rabbits were anesthetized with isoflurane (1–2% with 100% oxygen) using a face mask. Hearts were excised and perfused in a Langendorff apparatus using Krebs-Ringer solution [12].

### Mitochondrial Isolation, Labeling, and Measurement of ATP Content

For the imaging studies, mitochondria were isolated from human adult cardiac fibroblasts maintained in Fibroblast Medium-2 containing fetal bovine serum, fibroblast growth supplement-2, and antibiotic (penicillin/streptomycin) solution according to the supplier’s directions (ScienCell, Carlsbad, CA) [13, 14, 16]. Cardiac fibroblasts at 80% confluence were collected by trypsin digestion from 2 x 100 mm culture plates. Cells were centrifuged at a relative centrifugal force (RCF) of 300 for 10 minutes at room temperature and resuspended in 5 mL of 4°C homogenization buffer (300 mmol/L sucrose, 10 mmol/L HEPES-KOH, 1 mmol/L EGTA-KOH, pH 7.4) before being transferred to a C tube (Miltenyi Biotec, Cambridge, MA). Cells were homogenized using a GentleMACS Dissociator (Miltenyi Biotec, Cambridge, MA) using the ‘h-mito tissue-1’ pre-set program. The homogenate was incubated on ice for 10 minutes with 1 mg Subtilisin A protease from *Bacillus licheniformis* (Sigma-Aldrich, St. Louis, MO). The digested homogenate was serially filtered through 2 x 40 μm Falcon Cell Strainers (Thermo-Fisher, Waltham, MA) and 1 x 10 μm pluriSelect mesh (PluriSelect, San Diego, CA) that was saturated with ice cold homogenization buffer. Mitochondria were collected by centrifuging the filtrate at 8,476 RCF at 4°C for 10 minutes and resuspended in homogenization buffer [16]. Mitochondrial number was determined by hemocytometry [14]. Subsequently, human fibroblast mitochondria were labeled with 0.2 MBq ^18^F-R6G for 10 minutes at 0°C and then washed 3 times in homogenization buffer [15, 16]. For some experiments, fibroblast mitochondria were first cross-linked at 0°C to 10 mg *N*-succinimidyl ester-functionalized 30 nm magnetic iron (II,III) oxide particles (Sigma-Aldrich, St. Louis, MO) in homogenization buffer for 10 minutes followed by 5 washes in the same buffer containing 1 mg/mL fraction V bovine serum albumin (Sigma-Aldrich, St. Louis, MO). The ATP concentration in unlabeled and iron-labeled liver mitochondria was determined in the presence of 1 μmol/L adenosine diphosphate (ADP) substrate using the ATPlite Luminescence Assay System (Perkin Elmer, Waltham, MA) [14, 16].

### Mitochondrial Transplantation into Ischemic Hearts

Labeled mitochondria were used immediately for delivery into isolated heart preparations or centrifuged and placed in fixative for transmission electron microscopy [13]. For the imaging studies, isolated rabbit hearts were retrograde perfused for 10 minutes and then subjected to 30 minutes of global or regional ischemia. Global ischemia was induced by cross-clamping the aorta [12], while regional ischemia was achieved by passing 3–0 silk thread around the left anterior descending (LAD) coronary artery using a taper needle. Each end of the thread was then passed through a small vinyl tube to form a snare. The LAD artery was temporarily occluded by pulling the snare and fastening the tube with a mosquito clamp [12]. In globally ischemic hearts (n = 6) flow was restored to the heart by removing the cross-clamp. In regional ischemic hearts (n = 6), the snare was released to re-establish LAD arterial flow. Immediately after either global or regional ischemia, the rabbit hearts received 1 x 10^8^ human mitochondria labeled with ^18^F-R6G ± iron oxide nanoparticles by direct injection into the left ventricular free wall (n = 3) or through antegrade perfusion through the coronary arteries via aortic root (n = 3). For the injected heart preparations, mitochondria were resuspended in 0.8 mL homogenization buffer and delivered as eight 0.1 mL injections in the ischemic region using a 1 mL tuberculin syringe fitted with a 28 G needle [12]. For the perfused heart preparations, mitochondria were resuspended in 25 mL of 37°C Krebs-Ringer solution and administered antegrade to the coronary arteries through the aortic cannula over 1.5 minutes. Perfusion effluent was collected for the duration of reperfusion and counted using a CRC-15R Enhanced Dose Calibrator (Capintec, Ramsey, NJ) to assess incorporation of ^18^F-R6G-labeled mitochondria in each heart.

### Imaging of Transplanted Mitochondria in Ischemic Hearts

Rabbit hearts were perfusion fixed under constant pressure using 4% paraformaldehyde in phosphate buffered saline (PBS) (pH 7.4) and immediately imaged by PET and μCT using an Albira PET/SPECT/CT Preclinical Imaging System (Bruker, Billerica, MA) [19]. The LAD artery ligation site in regionally ischemic hearts was marked with Ethicon 5–0 surgical steel suture (Johnson & Johnson, New Brunswick, NJ). PET acquisition was performed for 1 hour followed by a 15 minute μCT acquisition using the Albira ‘best setting’ (*i*.*e*. 600 projections, 125 μm isotropic voxel size) at high dose (400 μA) and high voltage (45 kVp). In some instances, hearts were perfused with FITC-conjugated *Lycopersicon esculentum* lectin (Sigma-Aldrich, St. Louis, MO) prior to fixation. Hearts injected or perfused with dual-labeled (*i*.*e*. ^18^F-R6G and iron oxide) mitochondria were imaged on a BioSpec 70/30 USR 7T MRI System (Bruker Biospin, Billerica, MA). High-resolution 3-D T2-weighted images were acquired using a Rapid Acquisition with Relaxation Enhancement (RARE) sequence with a field of view (FOV) of 38 mm, 3-D isotropic resolution of 0.148 mm and fat suppression was applied. The RARE factor was 16, the effective echo time (TE) was 45.85 ms, repetition time (TR) was 1500 ms, number of averages was 1, and the total acquisition time was 1 hour and 42 minutes.

### Cardiac Tissue Histology and Microscopy

Ventricles from fixed hearts were embedded in paraffin and sectioned for histological examination [12, 13, 17, 20]. Rehydrated tissue sections (approximately 5 μm thickness) were subjected to antigen retrieval in 1 mmol/L EDTA prior to incubation with the following primary antibodies (Abcam, Cambridge, MA): anti-mitochondria mouse monoclonal antibody [MTC02] (ab3298), anti-MTCO2 rabbit monoclonal antibody [EPR3314] (ab79393), anti-MTCO2 rabbit polyclonal antibody (ab91317), anti-mitochondria mouse monoclonal antibody [113–1] (ab92824), and anti-MTCO2 mouse monoclonal antibody [12C4F12] (ab110258), anti-desmin rabbit monoclonal antibody (ab32362), and anti-sarcomeric α-actinin rabbit monoclonal antibody (ab68167) [12, 13, 20]. An anti-*Aspergillus niger* glucose oxidase (X0931) hybridoma cell culture supernatant (Dako, Carpinteria, CA) was used as an IgG_1_ negative control antibody as this protein is not expressed in mammalian tissues. These primary antibodies were detected with species-appropriate secondary antibodies conjugated to Alexa Fluor 488 or 568 dyes (Thermo-Fisher, Waltham, MA). Other sections were stained with Texas Red X-wheat germ agglutinin (WGA) and/or 4',6-diamidino-2-phenylindole (DAPI) (Thermo-Fisher, Waltham, MA) according to the manufacturer’s directions [13]. Mounted sections were visualized using a FSX100 inverted fluorescence microscope (Olympus, Tokyo, Japan) [14, 17]. Brightfield microscopy of sections stained using the Iron Stain Kit (Sigma-Aldrich, St. Louis MO) was performed with the same microscope. Isolated mitochondria were fixed in 1.25% formaldehyde, 2.5% grade I glutaraldehyde, and 0.03% picric acid in 100 mmol/L cacodylate buffer at 4°C and imaged by transmission electron microscopy [13].

### Contractile Function and Infarction Measurements

A separate group of rabbit hearts was subjected to either no injury (Sham group, n = 4) or 30 minutes regional ischemia followed with 120 minutes reperfusion (n = 10) as previously described [12]. These hearts were paced through the right atria at 80 ± 5 beats per minute using a coaxial bipolar stimulation electrode connected to a S48 electrical stimulator (Grass Technologies, Rockland, MA). Segmental shortening was measured by three 1.0 mm piezoelectric ultrasonic transducers (Sonometrics, London, Canada) implanted in the sub-endocardial layer of the ischemic area at risk (AAR) spaced approximately 10 mm apart [21]. Autologous mitochondria were isolated from rabbit liver tissue as described above and previously [16]. Following 30 minutes of regional ischemia, the snare was released and 25 mL of Krebs-Ringer solution (Control group; n = 5) or 25 mL of Krebs-Ringer solution containing 1 x 10^8^ unlabeled liver mitochondria (Mitochondria-treated group; n = 5) was delivered antegrade to the coronary arteries through the aortic root over 1.5 minutes. The hearts were then reperfused for 120 minutes. Sham hearts (n = 4) were not subjected to ischemia-reperfusion injury nor were they transplanted with mitochondria. Myocardial global and regional function was determined as described previously [12]. Regional contractility was measured as segmental shortening and expressed as a percentage of pre-ischemic segmental shortening. Quantitation of the AAR was accomplished using planimetry of Monastryl blue pigment (Sigma-Aldrich, St. Louis, MO) distribution after re-ligation of the LAD artery at the end of reperfusion [12]. Hearts were rapidly removed and sliced across the long axis of the left ventricle, from apex to base, into 0.5 cm thick transverse sections. These sections were incubated in 1% triphenyl tetrazolium chloride (TTC) (Sigma-Aldrich, St. Louis, MO) in PBS (pH 7.4) at 37°C for 20 minutes and then fixed for 24 hours in 10% neutral-buffered formalin solution [12, 13]. Afterward, sections were traced onto a clear acetate sheet over a glass plate and infarct size was assessed using planimetry [12, 13]. A separate set of Sham, Control, and Mitochondria-treated hearts were used for histological staining with the Artisan Masson’s Trichrome Stain kit (Dako, Carpinteria, CA). Control and Mitochondria groups were either injected or perfused with vehicle (*i*.*e*. Krebs-Ringer solution) or 1 x 10^8^ autologously-derived liver mitochondria in Krebs-Ringer solution, respectively.

### Statistical Analyses

Mitochondrial labeling and position in electron and fluorescent micrographs was assessed by two blinded observers. For iron labeling, 500 mitochondria from transmission electron micrographs were evaluated for the number of 30 nm nanoparticles attached to the outer membrane. The position of injected or perfused mitochondria in tissue sections was determined in slides stained with α-actinin, desmin, WGA, or *Lycopersicon esculentum* lectin and one of the anti-mitochondrial antibodies listed above. Micrographs were later analyzed for mitochondrial number and position using ImageJ software (National Institutes of Health, Bethesda, MD) [22]. Slides were derived from a minimum of three different rabbits in each regionally ischemic group and a total of 3,634 or 5,118 mitochondria were assessed in tissue sections from injected or perfused hearts, respectively. Values from these analyses are presented as percentage ± standard error of the mean (SEM). Global and local myocardial functional data was analyzed using repeated measures of a mixed model ANOVA to compare the effects of mitochondria delivered through coronary infusion (SAS, Cary, NC). The statistical approach included a compound symmetry correlation structure to account for multiple measurements within the same animal as determined using Akaike’s Information Criterion. A one-way ANOVA was employed for heart rate, AAR, and infarct size analyses and values represent mean ± SEM. A p < 0.05 was considered significant [12].

## Results

**Fig 1:**
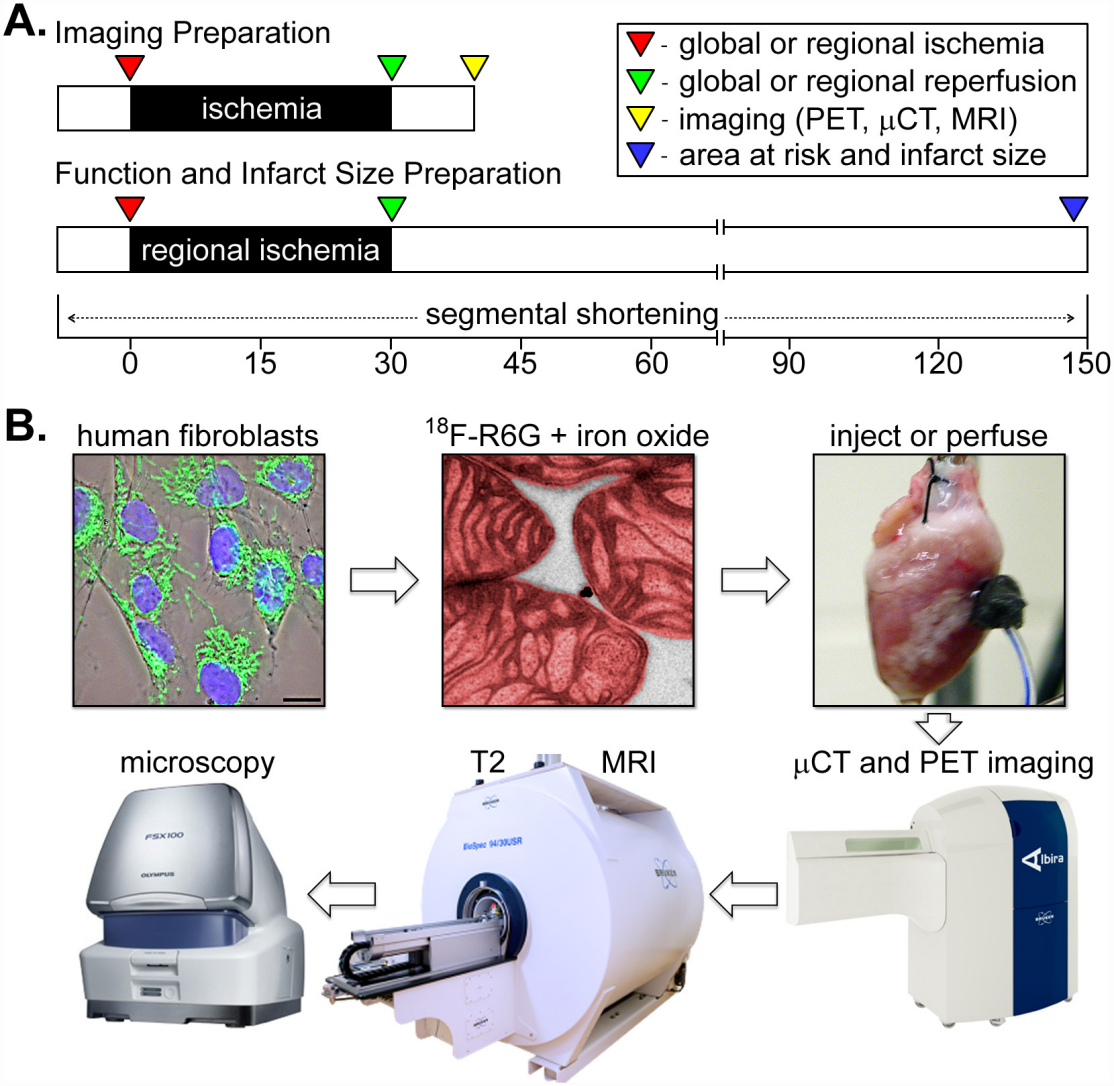
A schematic representation of the experimental procedures. (A) For imaging of mitochondrial distribution, the ischemic interval was 30 minutes followed by 10 minutes of reperfusion (top). Hearts used for functional and infarction measurements were subjected to 30 minutes of ischemia followed by 2 hours of reperfusion (bottom). In both instances, mitochondria were delivered to the heart at the onset of reperfusion. (B) Cultured human cardiac fibroblasts (fluorescently stained in this phase contrast image overlay with TOMM20 [green] to show mitochondria and DAPI [blue] to show nuclei), were used to isolate and label mitochondria with ^18^F-R6G (colorized as red in this transmission electron micrograph) and 30 nm iron oxide particles (black dots on the mitochondrial outer surface). Dual-labeled mitochondria were injected or perfused into ischemic Langendorff-perfused isolated hearts, which were imaged by PET and μCT followed with MRI. Hearts were then fixed, embedded, sectioned, and histologically stained for fluorescence and brightfield microscopy.

We designed this study to compare the acute distribution of exogenously-derived mitochondria delivered to the injured myocardium through either direct injection or coronary perfusion using several clinically-relevant imaging modalities (Fig 1). In isolated rabbit heart preparations subjected to global or regional ischemia, we discovered mitochondrial transplantation through the cardiac vasculature was practicable and occurred within minutes. Based on these findings, we expanded our study to include a functional assessment of contraction in regionally ischemic hearts treated with mitochondria infused through the coronary vasculature (Fig 1). By combining PET, μCT, and MRI with histological evaluation of engraftment as well as measurements of contractility and tissue viability, we have provided persuasive evidence that intracoronary delivery of respiration-competent mitochondria was a feasible and effective means to protect injured hearts from ischemia-reperfusion injury.

**Fig 2:**
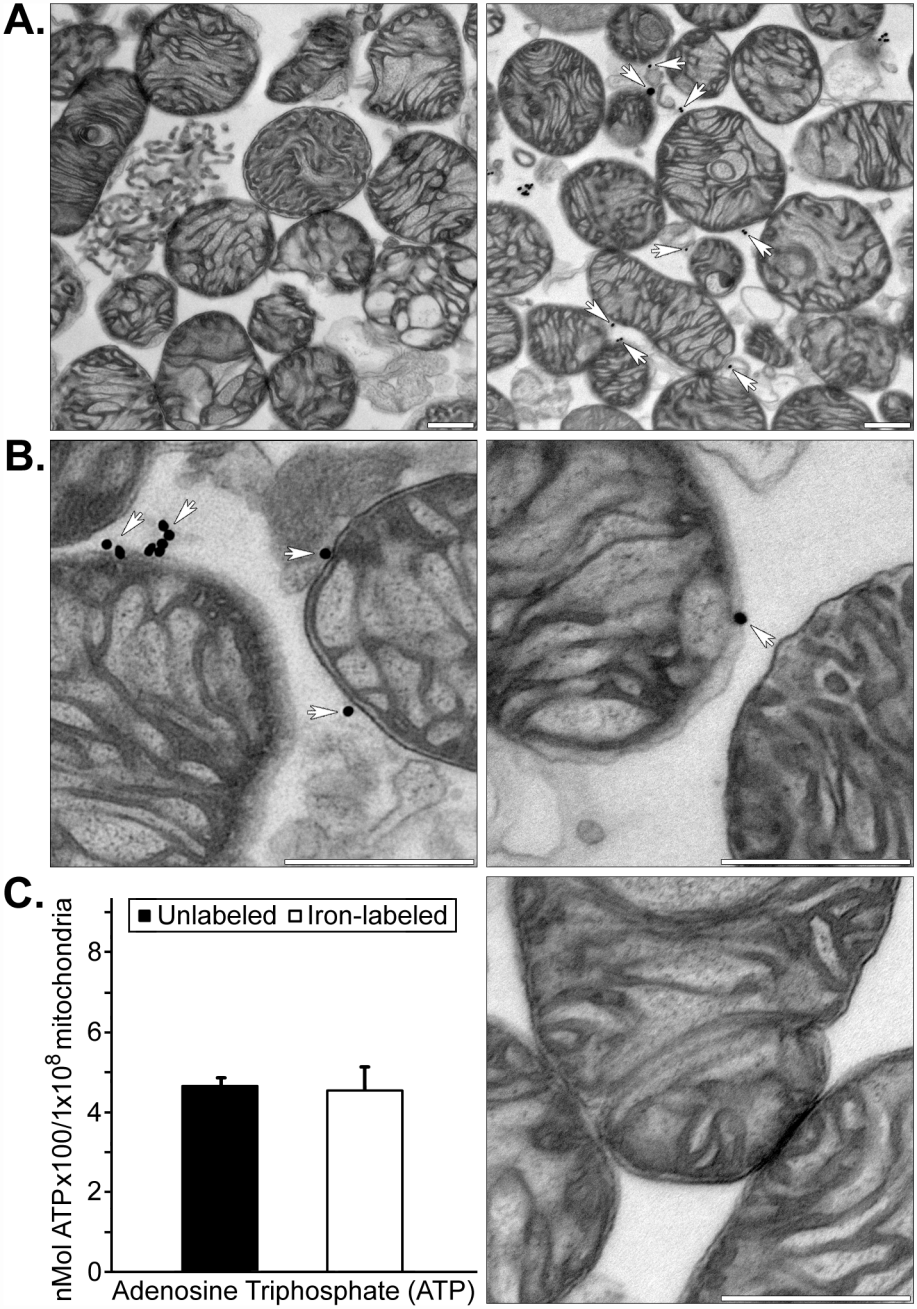
Transmission electron microscopy of iron oxide-labeled human mitochondria and ATP concentrations in labeled versus unlabeled isolated mitochondria. (A) Representative low magnification images of unlabeled adult human cardiac fibroblast mitochondria (left panel) and iron oxide-labeled mitochondria (right panel). (B) Representative high magnification images of 30 nm magnetic iron oxide particles associated with the outer mitochondrial membrane are indicated with arrows. Scale bars equal 500 nm. (C) ATP concentrations in unlabeled and iron oxide nanoparticle-labeled isolated human cardiac fibroblast mitochondria (left). A high magnification image of unlabeled mitochondria is shown at the right and the scale bar equals 500 nm. Isolated mitochondria were electron dense and ≤ 1% appeared fractured or damaged, regardless of whether they were unlabeled or cross-linked to iron oxide nanoparticles.

Some of the human mitochondria used for these imaging studies were labeled with both ^18^F-R6G and magnetic iron oxide nanoparticles to allow for their detection with PET as well as MRI. While it has been demonstrated that ^18^F-R6G specifically targets actively respiring mitochondria and that these organelles can be cross-linked to an amine-reactive fluorescent probe, we wanted to establish the extent and site of iron oxide labeling [14, 15]. We found isolated human mitochondria were labeled with relatively few 30 nm iron particles (4.10 ± 0.45, mean ± SEM) and these were attached to exposed amine groups on the outer membrane (Fig 2). By extensively washing the mitochondria in homogenization buffer containing bovine serum albumin, we were able to efficiently remove iron nanoparticles not covalently linked to the outer mitochondrial membrane because this protein has 59 lysine residues—30 to 35 of which have primary amine residues that are available to react with N-hydroxysuccinimidyl esters. Transmission electron microscopy also showed the mitochondria were largely intact and electron dense with conspicuous cristae, which is indicative of their viability. This assumption was supported by measurements of ATP content in unlabeled and iron oxide nanoparticle-labeled isolated mitochondria, which showed no significant differences in the concentration of this nucleoside triphosphate (Fig 2C).

**Fig 3:**
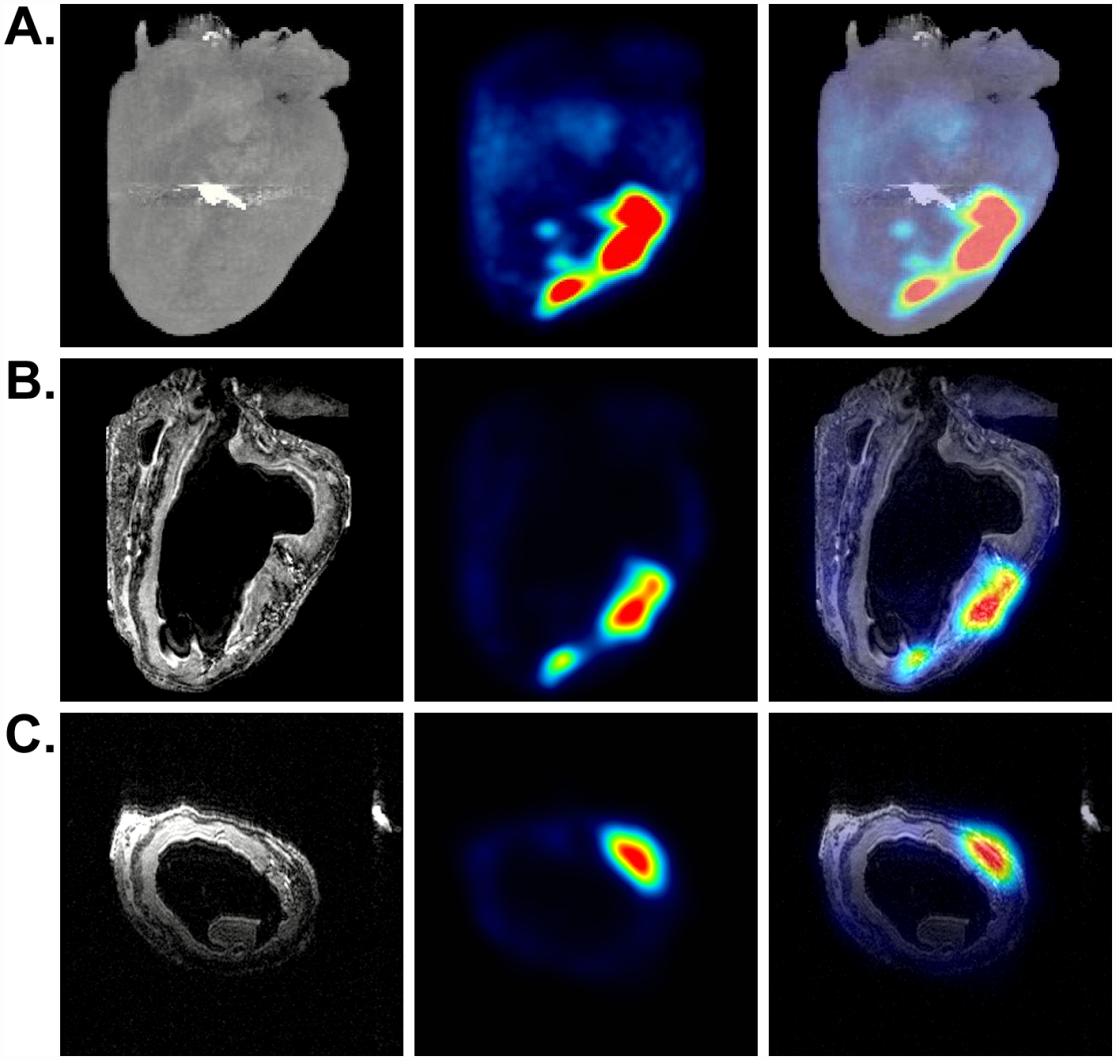
Imaging of a regionally ischemic heart injected with dual-labeled human mitochondria. (A) μCT, PET, and merged volumetric renderings are shown from left to right. The metal suture (bright signal on μCT) indicates the site of LAD coronary artery ligation. (B) Coronal slices of MRI (0.148 mm thickness) along with the corresponding PET scan as well as the merged MRI and PET images are shown (left to right). (C) Transverse slices of MRI, PET, and the merged images (left to right). Regions of hypointense T2-weighted MRI signals from iron correlate with the PET signals from ^18^F-R6G.

We introduced 1 x 10^8 18^F-R6G-labeled mitochondria into the left ventricular AAR in globally and regionally ischemic hearts and observed discrete PET signals corresponding to the injection sites (Fig 3 and S1 Fig). In ensuing experiments, we used mitochondria loaded with ^18^F-R6G and covalently decorated with iron oxide nanoparticles (Fig 2). In Fig 3, representative μCT (A, left panel), PET (A-C, middle panels), and MRI (B and C, left panels) images of a regionally ischemic heart are displayed. In addition to delineating the position of the ^18^F PET tracer signal in relation to the heart as a whole and the site of LAD coronary artery ligation (Fig 3A, right panel), these images show co-localization of ^18^F-R6G and the T2-weighted MRI signal resulting from the presence of magnetic iron (Fig 3B and 3C, right panels). The MRI signals produced using a RARE pulse sequence appear dark, which can be confused with other hypointense areas; however, no such signal was observed in the ventricular wall of control hearts (S2 Fig) [17]. Although we noticed comparable tracer signals from the injection of ^18^F-R6G-labeled mitochondria in both globally and regionally ischemic hearts (Fig 3 and S1 Fig), we occasionally observed a signal corona by PET and MRI (Fig 3 and S1 Mov), which suggested some level of mitochondrial dispersal outside the vicinity of the injection sites. Decay-corrected measurements of ^18^F-R6G using a dose calibrator revealed most of the ^18^F-R6G-labeled mitochondria remained contained within the injected hearts throughout reperfusion (77.3% ± 5.5, mean ± SEM).

**Fig 4:**
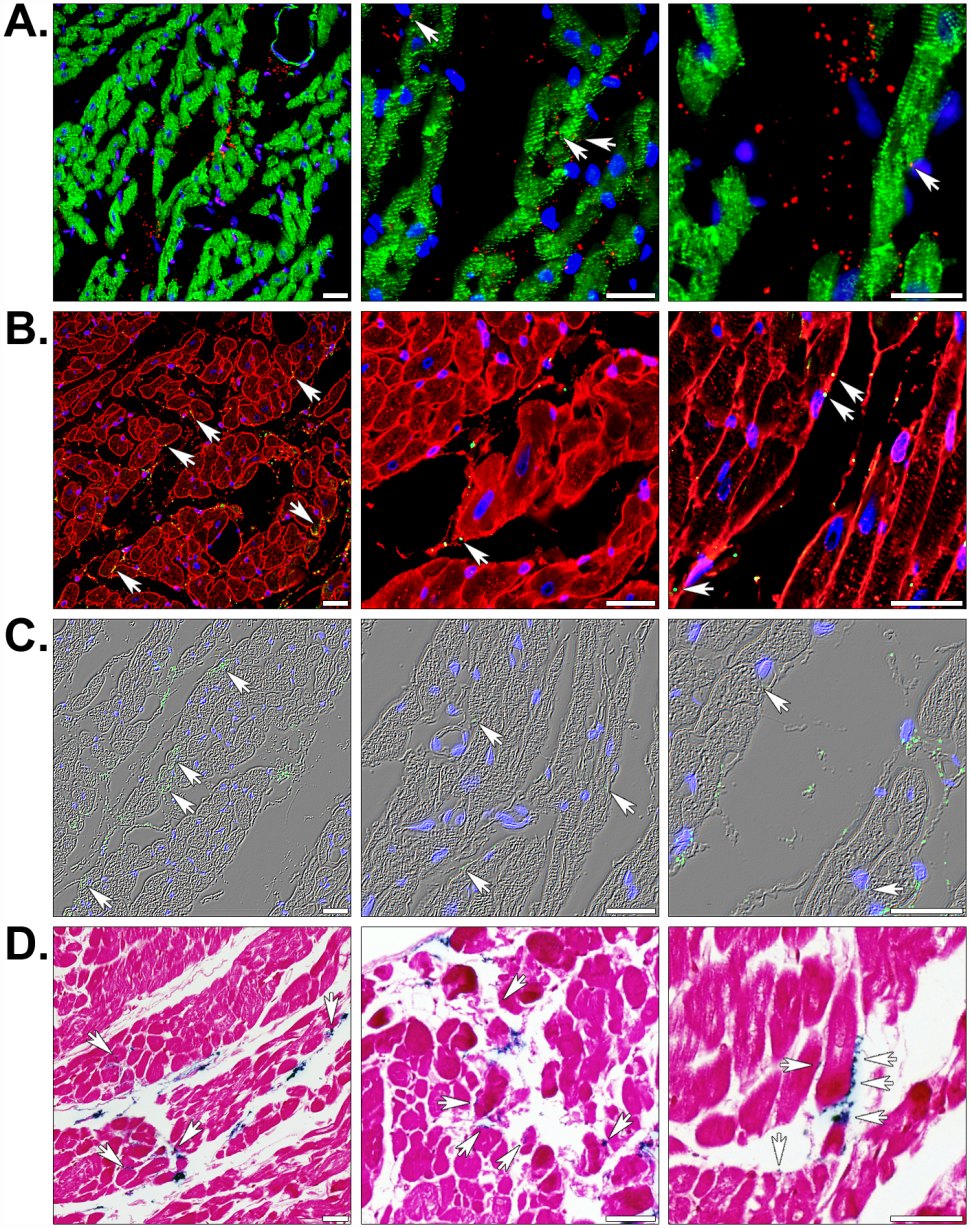
Histology of globally or regionally ischemic rabbit hearts injected with human mitochondria. (A) Injected heart sections were fluorescently stained for the muscle markers desmin (green), the human-specific mitochondrial marker MTCO2 (red), and nuclei using the DNA stain DAPI (blue). (B) Fluorescent staining with the membrane marker WGA (red), the 113–1 human mitochondrial marker (green), and DAPI (blue). (C) MTCO2 and nuclear staining is shown with phase contrast illumination. (D) Prussian blue (blue) and pararosaniline (pink) staining of injected mitochondria labeled with magnetic iron oxide nanoparticles. Scale bars represent 25 μm. Transplanted mitochondria associated with cardiac myocyte sarcolemmata are indicated (arrows).

To confirm the presence of human cardiac fibroblast-derived mitochondria at or near these injection sites, we immunofluorescently stained heart sections using an array of organelle-specific antibodies capable of distinguishing transplanted mitochondria from endogenous mitochondria (Fig 4 and S3 Fig). In the context of rabbit heart tissue, all of these antibodies specifically reacted with human mitochondria; however, the polyclonal antibodies (ab79393 and ab91317) showed the lowest signal-to-noise ratios. It is also worth noting that the serial sections from an injected heart (S3 Fig) contained different numbers of mitochondria in various positions attributable to the thickness of the paraffin sections (approximately 5 μm) and the small size of these organelles (diameters typically range from 250 to 1000 nm) [16]. The site of contraction band necrosis in S3 Fig was also indicative of the tissue being derived from the AAR. In Fig 4A, increasing magnifications of an injected heart illustrated the presence of a majority of mitochondria (shown in red) within the interstitial spaces between cardiomyocytes, which were identified by desmin (shown in green) or sarcomeric α-actinin staining (see below). In all images, nuclei are marked by DNA staining (blue) and mitochondria associated with cardiac muscle cells are indicated with arrows. Staining of the sarcolemma in tissue sections using WGA (Fig 4B) established co-localization of some injected mitochondria with cardiomyocytes. By overlaying nuclear and mitochondrial staining on phase contrast illuminated images (Fig 4C), we were able to get a better sense of the overall position of these organelles in the tissue. In addition, Prussian blue and pararosaniline staining confirmed that regions of tissue containing mitochondria were positive for iron in serial sections (Fig 4D). Together, our data indicate that despite the fact that the majority of mitochondria appeared to remain in the interstitial spaces minutes after injection, a significant proportion also appeared to be closely associated with cardiomyocytes. Enumeration of mitochondria in injected hearts revealed that 43.52% ± 4.46 (mean ± SEM) were attached to or found within cardiomyocytes.

**Fig 5:**
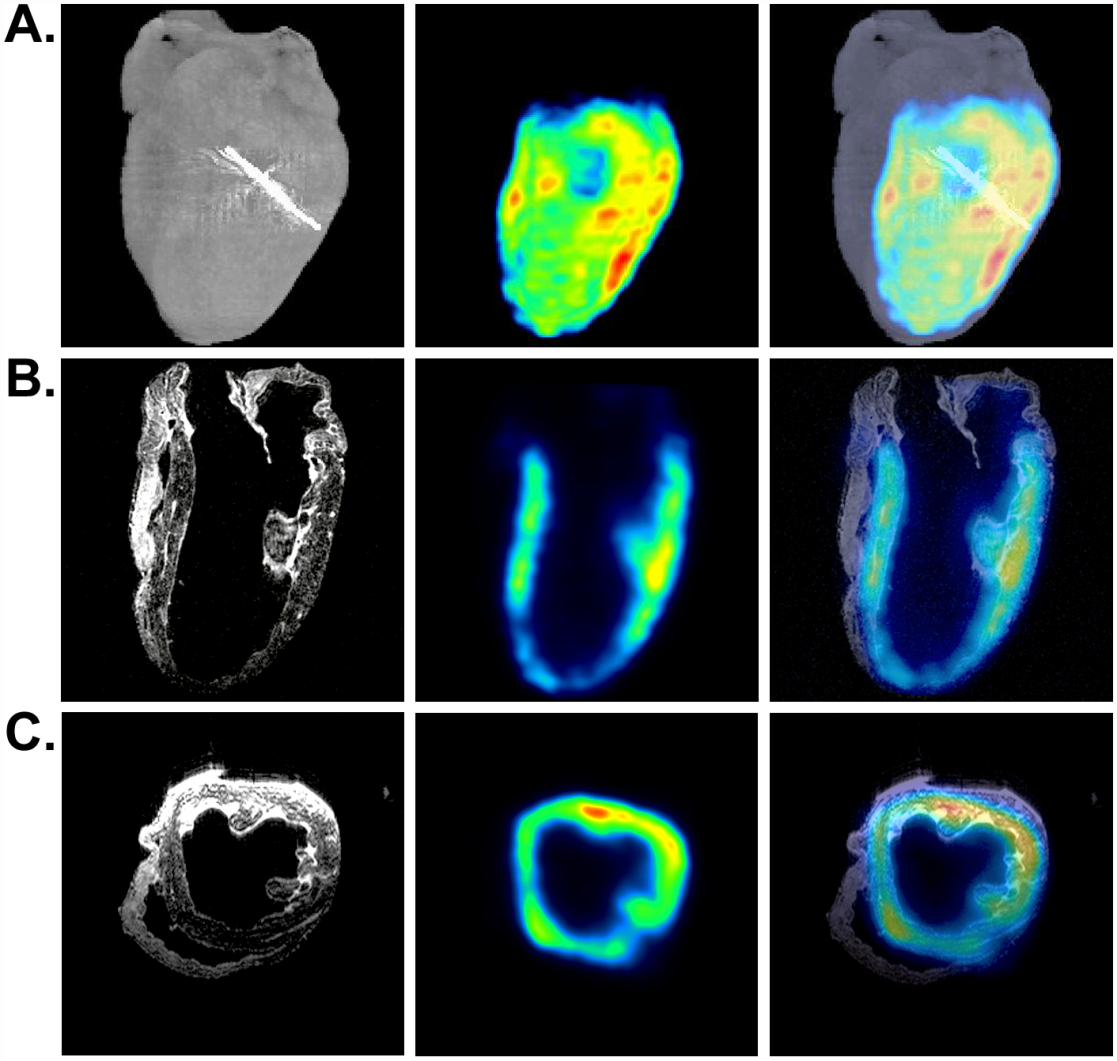
Imaging of a regionally ischemic heart perfused with dual-labeled human mitochondria. (A) Volumetric renderings of μCT, PET, and the merged acquisitions are shown (left to right). The μCT signal from the metal suture indicates the site of LAD coronary artery ligation. (B) Coronary slices from MRI (0.148 mm thickness) along with the corresponding PET scan as well as the merged MRI and PET images are depicted (left to right). (C) Transverse slices of MRI (0.148 mm thickness), PET, and the merged images (left to right) from the same perfused heart. Regions of hypointense T2-weighted MRI signals from iron correlate with PET signals from ^18^F-R6G.

To visualize exogenously-derived mitochondrial tissue distribution following infusion through the coronary vasculature rather than through direct injection, we perfused 1 x 10^8 18^F-R6G-labeled or ^18^F-R6G and iron oxide nanoparticle-labeled human mitochondria through the aorta in regionally (Fig 5) and globally ischemic (S1B Fig) isolated rabbit hearts. As described for Fig 3, hearts were imaged using μCT (A, left panel), PET (A-C, middle panels), and MRI (B and C, left panels). The volumetric rendering of the regionally ischemic heart shown in Fig 5A revealed ^18^F signal from the left ventricle; yet, no signal from the right ventricle (S2 Mov). Examination of individual slices (Fig 5B and 5C) verified this observation, which illustrated the utility of combined μCT and PET imaging. On the other hand, the remaining hearts we analyzed revealed tracer signals from the right ventricle (S1B and S1C Fig and not shown). Once again, we confirmed the hypointense MRI signal from magnetic iron oxide nanoparticles overlapped with the PET signal. For instance, as observed by PET in Fig 5, no T2-weighted signal emanated from the right ventricle. For comparison, the heart depicted in S1C Fig demonstrated a lack of perfused mitochondria in the AAR of a regionally ischemic heart attributable to administration of ^18^F-R6G-labeled organelles prior to release of the LAD coronary artery snare. Similar to our observations from the injected hearts, decay-corrected radioactivity measurements on the effluent from perfused hearts established that the majority of ^18^F-R6G-labeled mitochondria remained in the hearts during reperfusion (76.18% ± 11.85, mean ± SEM).

**Fig 6:**
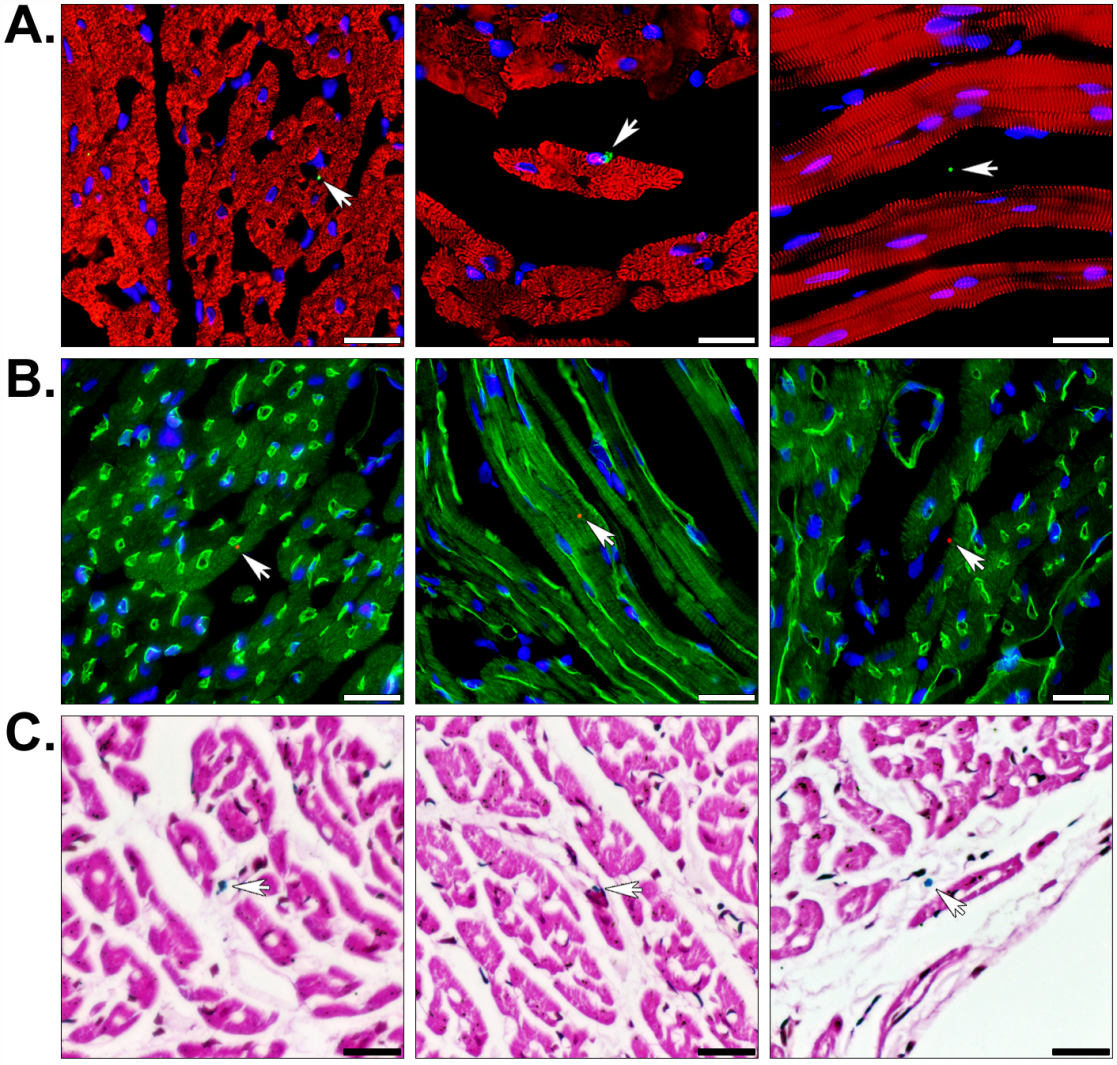
Histology of regionally ischemic hearts perfused with human mitochondria. (A) Perfused heart sections were stained with the muscle marker α-actinin (red) and the human mitochondrial marker MTCO2 (green) to show the position of transplanted mitochondria in the heart (arrows). (B) Some hearts were also perfused with FITC-lectin (green) prior to fixation to display luminal surfaces of blood vessels. These sections were counter-stained with the 113–1 human mitochondrial marker (red) and nuclei were stained with DAPI (blue). The staining shows transplanted mitochondria associated with the vasculature, within interstitial spaces, and attached to cardiomyocytes. (C) Prussian blue staining (blue) and a pararosaniline counterstain (pink) confirmed transplanted mitochondria were labeled with magnetic iron oxide nanoparticles. Scale bars equal 25 μm.

Because the PET and MRI experiments indicated that intracoronary infusion resulted in extensive and rapid dispersal of mitochondria throughout the heart, we examined stained tissue sections by brightfield and fluorescence microscopy. Species- and tissue-specific immunofluorescent staining revealed infused mitochondria were associated with blood vessels, cardiomyocytes, and the interstitium (Fig 6); but, generally did not appear in clusters as observed in the injected hearts (Fig 4). A quantitative assessment of perfused mitochondrial position in the tissue demonstrated 24.76% ± 2.50 (mean ± SEM) and 23.64% ± 2.42 (mean ± SEM) of these exogenous organelles were associated with cardiomyocytes and blood vessels, respectively. Intriguingly, our histological analyses indicated mitochondria appeared to extravasate within the 10 minute reperfusion phase and some of these organelles seemed to be internalized by cardiomyocytes [11, 13, 14]. In Fig 6, the left and middle images are representative examples of human mitochondria closely associated with capillaries and cardiomyocytes, respectively. The panels on the right side of the figure demonstrate infused mitochondria within the interstitial spaces. Again, Prussian blue staining verified that the dual-labeled mitochondria were attached to iron particles (Fig 6C). Because we were concerned with correctly identifying exogenous mitochondria in heart sections and their size approached the diffraction limit for diffraction limited optical microscopy, we validated our immunofluorescent staining using five different antibodies (S3 Fig) [23]. Each of these could distinguish human mitochondria from endogenous rabbit mitochondria in serial sections from an injected, globally ischemic rabbit heart.

**Fig 7:**
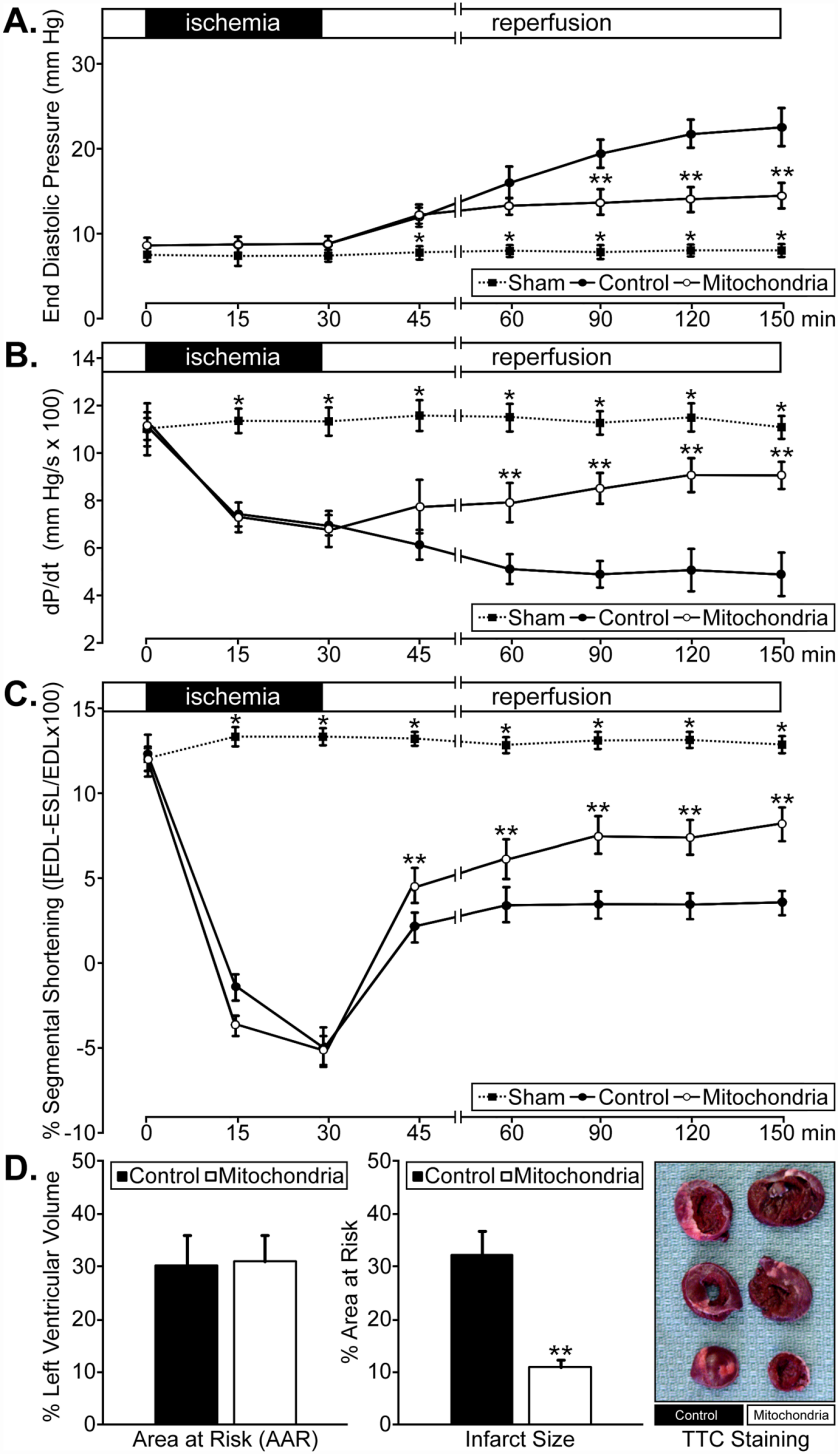
Myocardial function in regionally ischemic hearts perfused with autologous rabbit liver mitochondria. (A) End diastolic pressure (mm Hg); (B) positive dP/dt (mm Hg/s x 100); (C) % segmental shortening (end-diastolic length [EDL] minus end-systolic length [ESL] over end-diastolic length [EDL] x 100) in Control and Mitochondria heart groups, pre-ischemia, and during 30 minutes regional ischemia and 120 minutes of reperfusion. Sham groups were not subjected to ischemia and reperfusion or mitochondrial treatment. (D) AAR (% left ventricular volume) and infarct size (% AAR) following 30 minutes regional ischemia and 120 minutes reperfusion in Control and Mitochondria-treated hearts (left and middle, respectively). (A-D) * indicates a p < 0.001 between the Sham group and both Control and Mitochondria groups for EDP, dP/dt, and % segmental shortening at the indicated times. ** indicates a p < 0.001 between Control and Mitochondria-treated groups for EDP, dP/dt, and % segmental shortening at the indicated times as well as for infarct size at the end of reperfusion.

Given that intracoronary delivery of these organelles resulted in widespread myocardial dispersion, we assessed the functional consequences of this approach using autologously-derived liver mitochondria. Global and regional functional analyses demonstrated that there were no significant differences in end diastolic pressure (EDP, mm Hg), positive dP/dt (mm Hg/sec), or % segmental shortening prior to regional ischemia between Sham, Control, and Mitochondria groups (Fig 7). Concurrently, there were no significant differences in heart rate throughout pre-ischemia, ischemia, or reperfusion in Sham (82.88 ± 0.09 beats per minute, mean ± SEM) or Control (83.04 ± 0.13 beats per minute, mean ± SEM) versus Mitochondria-treated hearts (82.60 ± 0.18 beats per minute, mean ± SEM). With the onset of regional ischemia, dP/dt and % segmental shortening values were significantly decreased in both Control and Mitochondria groups at 15 (p < 0.001 for each) and 30 minutes (p < 0.001 for each) when compared to pre-ischemic values or non-ischemic Sham hearts (Fig 7A–7C). There were no significant differences observed between the Control and Mitochondria groups in dP/dT or % segmental shortening measurements at the end of ischemia (Fig 7B and 7C). During the 2 hour reperfusion interval, EDP was significantly decreased in the Mitochondria group compared with the Control group at 60, 90, and 120 minutes (*i*.*e*. 90, 120, and 150 minutes from the start of the experiment) (p < 0.001 for each) (Fig 7A). The % segmental shortening and dP/dt results were significantly increased at 30 (p < 0.001), 60 (p < 0.001), 90 (p < 0.001), and 120 (p < 0.001) minutes of reperfusion in the Mitochondria group when compared to Control hearts (Fig 7B and 7C). The % segmental shortening values at 15 minutes were also significantly different (p < 0.001) between these groups (Fig 7C). These results indicate that there was significantly enhanced global and regional myocardial function in injured hearts perfused with mitochondria compared to Control hearts; however, function was only partly restored toward pre-ischemic (p < 0.001) or non-ischemic values (p < 0.001). In addition, the AAR was 30% ± 5.7 (mean ± SEM) in Control hearts and 31% ± 4.4 (mean ± SEM) in Mitochondria-treated hearts (Fig 7D, left). There was no significant difference in AAR between these groups; however, we did detect a significant reduction (p < 0.001) in infarct size in the Mitochondria-treated hearts compared to the Control hearts (Fig 7D, middle and right).

Supplemental Fig 4 (S4 Fig) shows low magnification images of Masson’s trichrome stained Sham (A), Control (B), and Mitochondria-treated (C) rabbit heart tissues. Regionally ischemic hearts (B and C) were injected (left panels) or perfused (right panels) with Krebs-Ringer solution (vehicle) or autologous liver mitochondria. Examination of the morphology of vehicle-treated ischemic tissue sections (B) showed evidence of longitudinal and transverse interfibrillar separation compared with non-ischemic tissue sections (A) or those treated with mitochondria (C), which corroborates our earlier findings [19]. There were no detectable morphological differences between injected or perfused sections within any of these groups.

## Discussion

Our previous research established that cardiac ischemia detrimentally alters mitochondrial structure, volume, calcium accumulation, complex activity, oxygen consumption, and high energy phosphate synthesis [3–7, 11, 13, 21]. These events occur during ischemia and extend into reperfusion to severely compromise functional recovery of the heart and the viability of cardiac cells. We have demonstrated that the transplantation of autologously-derived mitochondria isolated from a remote, non-ischemic tissue source, significantly reduces infarct size and increases in tissue ATP content, cell viability, and post-ischemic contractile function [12, 13]. In these earlier studies, we directly injected structurally-intact, viable mitochondria into the ventricular free wall at the onset of reperfusion. A limitation of this method of delivery was the need for surgical access to the myocardium; so, we have assessed a less invasive means of mitochondrial delivery. In the present study, we provide evidence that vascular delivery of exogenous mitochondria through the coronary arteries results in the rapid and widespread distribution of these organelles throughout the myocardium. These findings contrast with parallel experiments using direct injection, which permitted higher concentrations of mitochondria to be delivered to targeted region in the myocardium [12, 13].

The transplanted mitochondria were typically located within interstitial spaces; yet, a number of these organelles were closely associated with blood vessels and cardiomyocytes. In some instances, we observed mitochondria that appeared to be internalized by cardiac muscle cells, despite the transient exposure to these organelles (10 minutes) [13, 14]. In our functional studies, we were able to show that mitochondrial delivery by vascular perfusion significantly enhanced global (dP/dt) and regional myocardial function (systolic shortening) during the 120 minute reperfusion interval following 30 minutes of regional ischemia. Infarct size measured at the end of reperfusion was also significantly decreased by vascular perfusion of autologous mitochondria. These findings are in agreement with our previous observations that injection of autologous mitochondria into the area at risk significantly enhanced myocardial function and significantly decreased myocardial infarct size [11]. A comparison between the two routes of delivery indicated that autologous mitochondrial transplantation is efficacious as a cardioprotective therapy whether these organelles are directly injected or infused through the coronary arteries. Consequently, we believe these studies represent an important step toward the clinical implementation of this novel therapeutic approach to enhance myocardial function and salvage the ischemic myocardium.

We employed several clinically-relevant imaging modalities to visualize the fate of human mitochondria delivered to globally or regionally ischemic rabbit hearts (Fig 1 and S1 Fig). By spatially registering images acquired using μCT and PET, we were able to accurately determine the anatomical location of the injected or perfused ^18^F-R6G-labeled mitochondria. By also labeling these organelles with 30 nm iron oxide particles (Fig 2), we were able to obtain more detailed information pertaining to transplanted mitochondrial distribution in the injured heart. Covalent attachment of iron oxide nanoparticles to exposed amine residues on the outer membrane ought to permit long-term tracking of mitochondria *in vivo* using MRI in ensuing studies. For example, we were able to monitor the engraftment of iron-labeled cells surgically implanted in the heart for up to one year using μCT and MRI [17]. While acute administration of mitochondria provides a significant functional improvement in ischemic hearts, non-invasively determining the disposition of these organelles over time will be important in establishing this treatment approach for other therapeutic applications directed at replacing impaired endogenous mitochondria and augmenting organ function [12, 14].

We recognize there are drawbacks associated with the use of T2-weighted MRI for monitoring engraftment of transplanted cells and organelles in the body [17]. Because iron oxide-based contrast agents result in signal loss during T2-weighted MRI, labeled regions can be difficult to distinguish from other areas of negative contrast and a quantitative assessment of the signal cannot be performed on very hypointense images [17]. Despite these shortcomings, we observed substantial overlap of PET and MRI signals in single image slices corresponding to dual-labeled mitochondrial injection sites (Fig 3) or areas of infusion (Fig 5) in the ventricular wall. Even in instances of quite diffuse ^18^F-R6G accumulation, such as that observed in the interventricular septum in Fig 3, we noted a corresponding hypointense MRI signal. Though the other hearts we used for injection of labeled mitochondria did not exhibit dispersal of tracer signal, we chose to present this example as it indicates the sensitivity of these imaging methods. We speculate the apparent spread of mitochondria in this heart was due to inadvertent injection into a circumferential vessel like the left circumflex artery. In any event, control hearts did not show equivalent hypointense signals by MRI (S2 Fig) nor did the right ventricle of the perfused heart shown in Fig 5. Lack of exogenous mitochondrial perfusion into the right ventricle in this particular heart was likely due to impairment of right coronary artery flow because the aorta was straightened by the cannula during retrograde perfusion. In addition, we did not observe hypointense signals resulting from edema in regionally ischemic control hearts (S2B Fig). It is possible that the administration of unlabeled mitochondria in these control hearts diminished the accumulation of interstitial fluid that is commonly observed following cardiac injury. Nevertheless, we are confident the regions of negative contrast observed by MRI in those hearts receiving iron oxide-labeled mitochondria represent the position of these organelles in tissue. When registered with PET data, it becomes clear that T2-weighted MRI signals correlate well with the position of the ^18^F-R6G tracer and may represent a more sensitive means to non-invasively detect transplanted mitochondria.

We verified the presence of mitochondria at cardiac injection sites (Fig 4) or throughout hearts perfused with these organelles (Fig 6) using in-depth histological analyses. For each heart, a minimum of 400 sections were analyzed for the presence of human mitochondria using a variety of species-specific antibodies (S3 Fig). Slides were fluorescently stained for muscle proteins (*e*.*g*. desmin or sarcomeric α-actinin), membrane glycoproteins on the cell surface (using WGA), and the lumen of perfused blood vessels (with *Lycopersicon esculentum* lectin). In hearts treated with dual-labeled mitochondria, we used Prussian blue staining to verify mitochondria remained conjugated to iron oxide nanoparticles. Collectively, our histological analyses enabled us to establish the precise position of transplanted mitochondria in myocardial tissues. In regionally and globally ischemic hearts, we found clusters of mitochondria corresponding to the injection sites (Fig 4) or discrete mitochondria throughout hearts infused with these organelles (Fig 6). Injected mitochondria were predominantly localized in the interstitium; however, a sizeable percentage of these were adjacent to or within cardiomyocytes (Fig 4). Exogenous mitochondria delivered through the coronary vasculature were spread throughout the heart, rather than concentrated in a particular region. Once again, individual mitochondria were observed within capillaries, cardiomyocytes, and interstitial spaces (Fig 6) and radioactive dose measurements of the coronary effluent proved the majority of infused mitochondria remained in the heart. In spite of these observations, the infused mitochondria would be greatly outnumbered by endogenous mitochondria; so, we were uncertain whether this delivery method would have a functional effect. On the other hand, our earlier findings proved injection of mitochondria into globally or regionally ischemic hearts provided a significant improvement in functional recovery and infarct size despite a similar concern [12, 13].

To determine the efficacy of vascular delivery of mitochondria, rabbit hearts were perfused with unlabeled, autologously-derived mitochondria in order to directly compare our findings to previous reports [13]. Cardiac functional analyses (Fig 7A–7C) revealed significant improvement in contractility in the ischemic region and globally during reperfusion (Fig 7B) [12]. Despite these functional improvements, ventricular contractility in mitochondria-treated hearts was never completely restored to the pre-ischemic or non-ischemic levels. We found vascular delivery of exogenous mitochondria also reduced infarct size (Fig 7D) similar to that previously observed by direct injection of mitochondria [12]. Unlike the preparations used for molecular imaging (Fig 1), we reperfused these hearts for 2 hours following intracoronary delivery of mitochondria. We found recovery of dP/dt and systolic shortening throughout the reperfusion interval beginning at 15 minutes, similar to that observed *in situ* [12, 13]. In addition, we observed attenuation in the rise of end diastolic pressure that results from ischemic injury to the myocardium.

Our previous studies demonstrated that the mechanisms through which mitochondrial transplantation provide for cardioprotection are mitochondrial-dependent [12, 13]. These studies showed that the requirement for viable mitochondria is paramount [12, 16]. Non-viable mitochondria, mitochondrial fractions (*e*.*g*. proteins and complex I-V), mitochondrial DNA or RNA, and exogenous ATP or ADP, when directly injected into the ischemic region during early reperfusion, failed to provide any significant cardioprotection or functional benefit [12]. Our studies have also demonstrated that once transplanted, exogenous mitochondria provide for cardioprotection both extracellularly and intracellularly [16]. Immediately following mitochondrial transplantation there was an increase in total tissue ATP content, augmented expression of mitochondrial proteins, and elaboration of precursor metabolites that support energy production and cellular respiration [13]. Transplanted mitochondria also amplified cytokine and chemokine expression levels associated with improved vascularization, protection against cardiomyocyte apoptosis, and enhanced cardiac functional recovery [13].

More recently, we showed that exogenous mitochondria are taken up by myocardial cells through an actin-dependent endocytotic mechanism [14]. Once inside cells, transplanted mitochondria significantly increased oxygen consumption rates and ATP synthesis resulting in rescue of cellular function. We also found transplanted mitochondria repaired damaged mtDNA [14]. In studies using HeLa ρ° cells, which are depleted of mtDNA and, therefore, incapable of oxidative phosphorylation, we showed that transplantation of mitochondria with intact mtDNA restored cellular respiration through oxidative phosphorylation and increased cellular ATP content. These studies proved that transplanted mitochondria repaired the damaged mtDNA of HeLa ρ° cells and remained in these cells for at least 3 weeks [14].

In the experiments described here, we determined that mitochondrial transplantation through coronary artery infusion is possible and provides for reduction in infarct size as well as enhancement of cardiac function in regionally ischemic hearts. While additional *in vivo* studies are needed, the ability to deliver mitochondria by vascular infusion has the potential to be applicable to several cardiovascular clinical procedures [11]. For example, during coronary artery bypass grafting, mitochondria could be injected into the ischemic region following bypass and just prior to aortic cross clamp removal. For percutaneous coronary interventions for ST-segment elevation myocardial infarction, isolated, exogenous mitochondria could be delivered to the heart during coronary revascularization.

## Conclusions

We demonstrated that mitochondria can be delivered to the ischemic heart through the vasculature and that transplantation of these organelles affords an important functional benefit. We believe our findings will help increase the clinical implementation of this technology for coronary artery bypass grafting and revascularization following myocardial infarction [11].

## Supporting Information

S1 Fig Imaging of globally and regionally ischemic hearts injected with 1 x 10^8^ dual-labeled human mitochondria. (A) Volumetric renderings of the μCT, PET, and the merged acquisition are shown from left to right. (B) Equivalent images from a globally ischemic heart perfused with the same number of dual-labeled cardiac fibroblast mitochondria. (C) A regionally ischemic heart perfused with 1 x 10^8^ dual-labeled mitochondria at the end of the ischemic interval prior to the removal of the snare to demonstrate signal exclusion from the AAR.
(TIF)

S2 Fig T2-weighted MRI of non-ischemic and ischemic rabbit hearts. (A) Coronal and transverse slices are shown (0.15 mm thickness). (B) The same views of a regionally ischemic rabbit heart perfused with unlabeled liver mitochondria. Hypointense regions within the ventricular walls were not observed in any image slices.
(TIF)

S3 Fig Histology of a regionally ischemic heart injected with human mitochondria. (A) Serial heart sections from an injection site were stained with sarcomeric α-actinin (red) and five different mitochondrial antibodies to show their specificity in detecting human mitochondria (green) in rabbit tissue. These antibodies were as follows: anti-mitochondria mouse monoclonal antibody [MTC02] (ab3298), anti-MTCO2 rabbit monoclonal antibody [EPR3314] (ab79393), anti-MTCO2 rabbit polyclonal antibody (ab91317), anti-mitochondria mouse monoclonal antibody [113–1] (ab92824), and anti-MTCO2 mouse monoclonal antibody [12C4F12] (ab110258) in addition to a negative control antibody. (B) The same images as described above are displayed with the red channel subtracted and the antibodies identified by catalog number (Abcam, Cambridge, MA). Nuclei stained with DAPI are also depicted in each image (blue). A cluster of transplanted mitochondria in an interstitial space are highlighted (dotted line) and a region of contraction band necrosis was also apparent (arrows). Scale bars equal 50 μm.
(TIF)

S4 Fig Representative Masson’s trichrome staining of tissue sections from hearts injected with autologous liver mitochondria. (A) Sham, (B) Control, and (C) Mitochondria experimental groups are shown. Non-ischemic (A) and regionally ischemic (B and C) rabbit hearts were injected (left panels) or perfused (right panels) with vehicle (B) or 1 x 10^8^ mitochondria (C). Scale bars equal 500 μm.
(TIF)

S1 Mov A longitudinal 360 degree rotation of the μCT, PET, and merged volumetric renderings of the injected heart shown in Fig 3. The metal suture (bright signal on μCT) indicates the site of LAD coronary artery ligation.
(MPG)

S2 Mov A longitudinal 360 degree rotation of the μCT, PET, and merged volumetric renderings of the perfused heart shown in Fig 5. The metal suture (bright signal on μCT) indicates the site of LAD coronary artery ligation.
(MPG)
