# Supplementary figures and images for: Intracoronary Delivery of Mitochondria to the Ischemic Heart for Cardioprotection
Source: PLoS One. 2016 Aug 8;11(8):e0160889. doi: 10.1371/journal.pone.0160889 (PMC4976938; doi:10.1371/journal.pone.0160889)

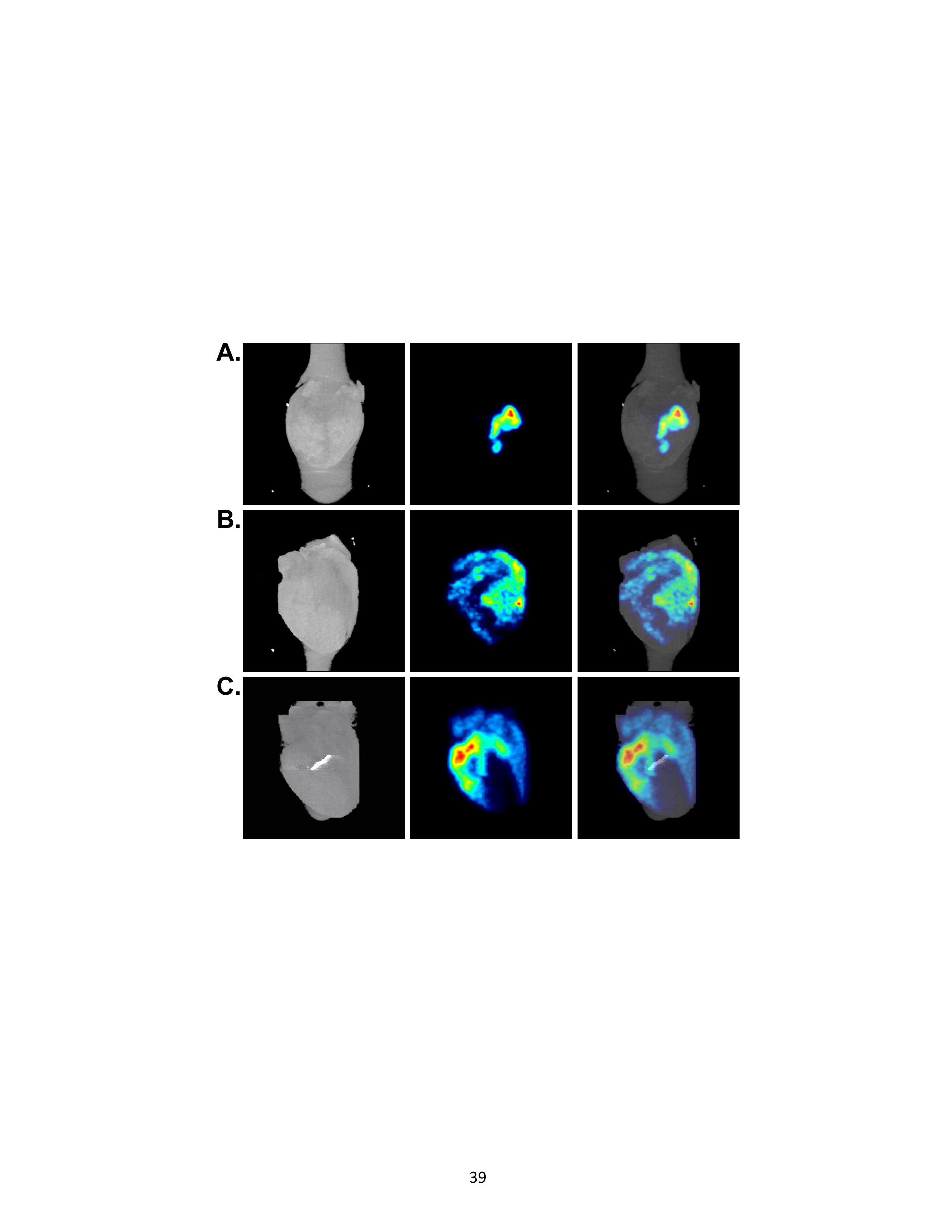

Supplement: S1 Fig — (A) Volumetric renderings of the μCT, PET, and the merged acquisition are shown from left to right. (B) Equivalent images from a globally ischemic heart perfused with the same number of dual-labeled cardiac fibroblast mitochondria. (C) A regionally ischemic heart perfused with 1 x 10^8^ dual-labeled mitochondria at the end of the ischemic interval prior to the removal of the snare to demonstrate signal exclusion from the AAR. (TIF) [file pone.0160889.s001.tif]

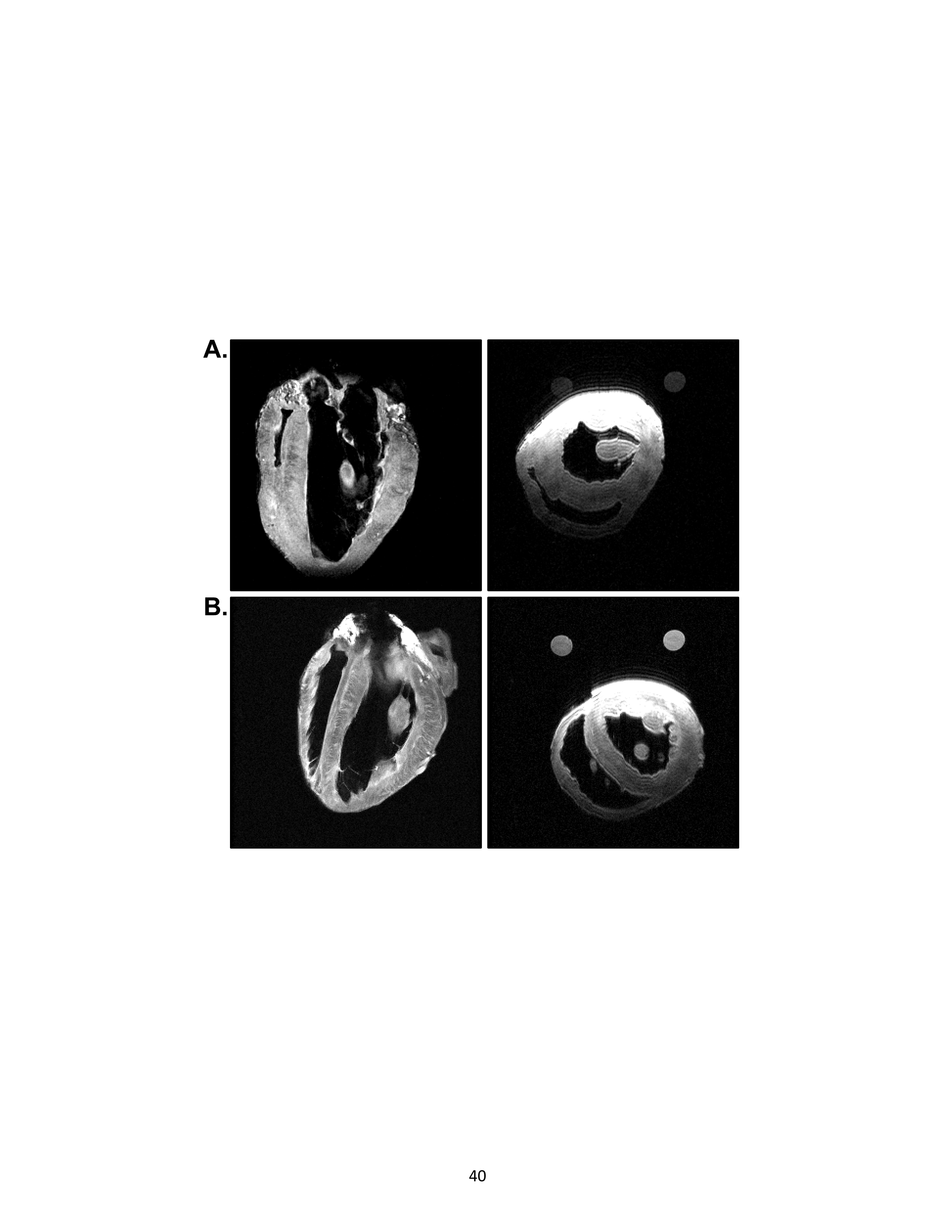

Supplement: S2 Fig — (A) Coronal and transverse slices are shown (0.15 mm thickness). (B) The same views of a regionally ischemic rabbit heart perfused with unlabeled liver mitochondria. Hypointense regions within the ventricular walls were not observed in any image slices. (TIF) [file pone.0160889.s002.tif]

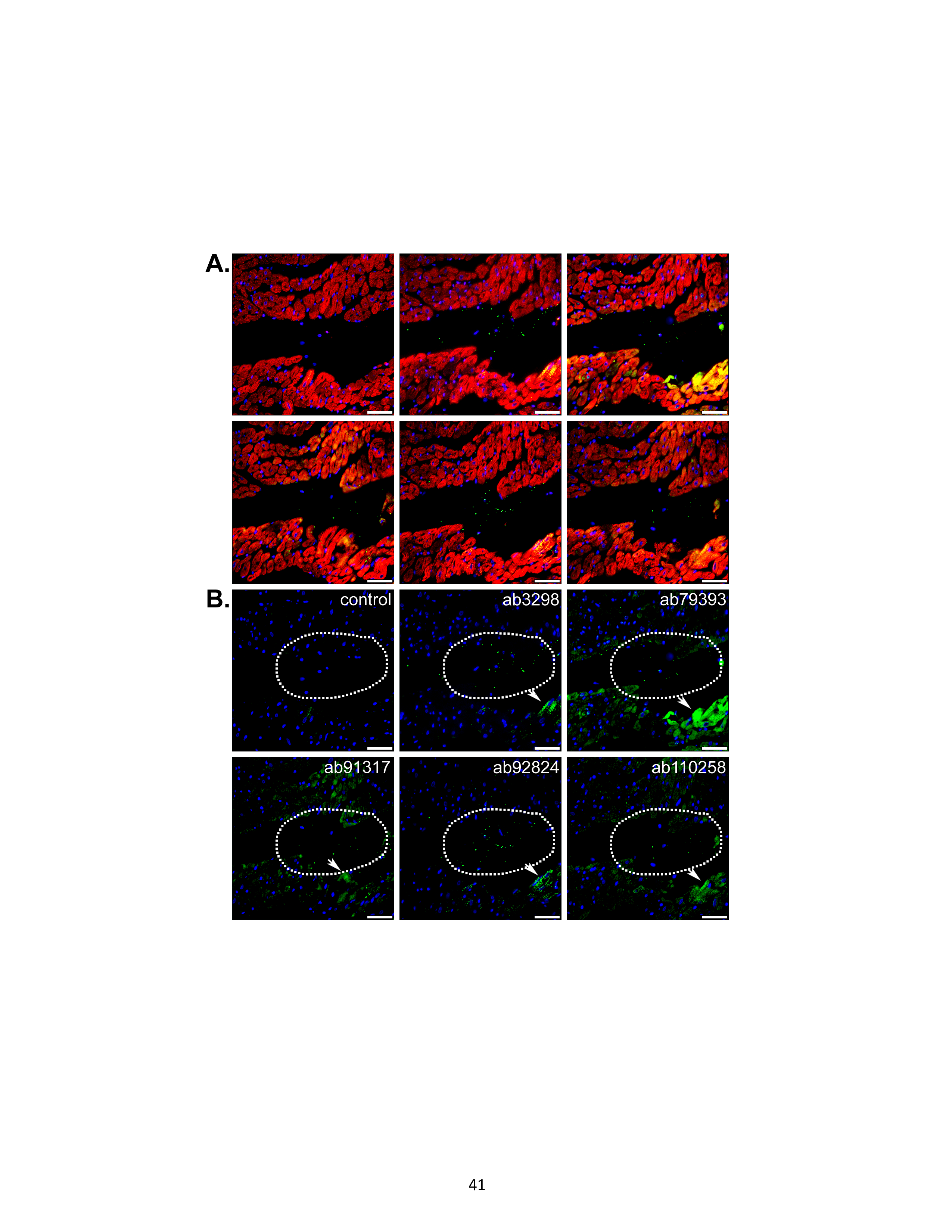

Supplement: S3 Fig — (A) Serial heart sections from an injection site were stained with sarcomeric α-actinin (red) and five different mitochondrial antibodies to show their specificity in detecting human mitochondria (green) in rabbit tissue. These antibodies were as follows: anti-mitochondria mouse monoclonal antibody [MTC02] (ab3298), anti-MTCO2 rabbit monoclonal antibody [EPR3314] (ab79393), anti-MTCO2 rabbit polyclonal antibody (ab91317), anti-mitochondria mouse monoclonal antibody [113–1] (ab92824), and anti-MTCO2 mouse monoclonal antibody [12C4F12] (ab110258) in addition to a negative control antibody. (B) The same images as described above are displayed with the red channel subtracted and the antibodies identified by catalog number (Abcam, Cambridge, MA). Nuclei stained with DAPI are also depicted in each image (blue). A cluster of transplanted mitochondria in an interstitial space are highlighted (dotted line) and a region of contraction band necrosis was also apparent (arrows). Scale bars equal 50 μm. (TIF) [file pone.0160889.s003.tif]

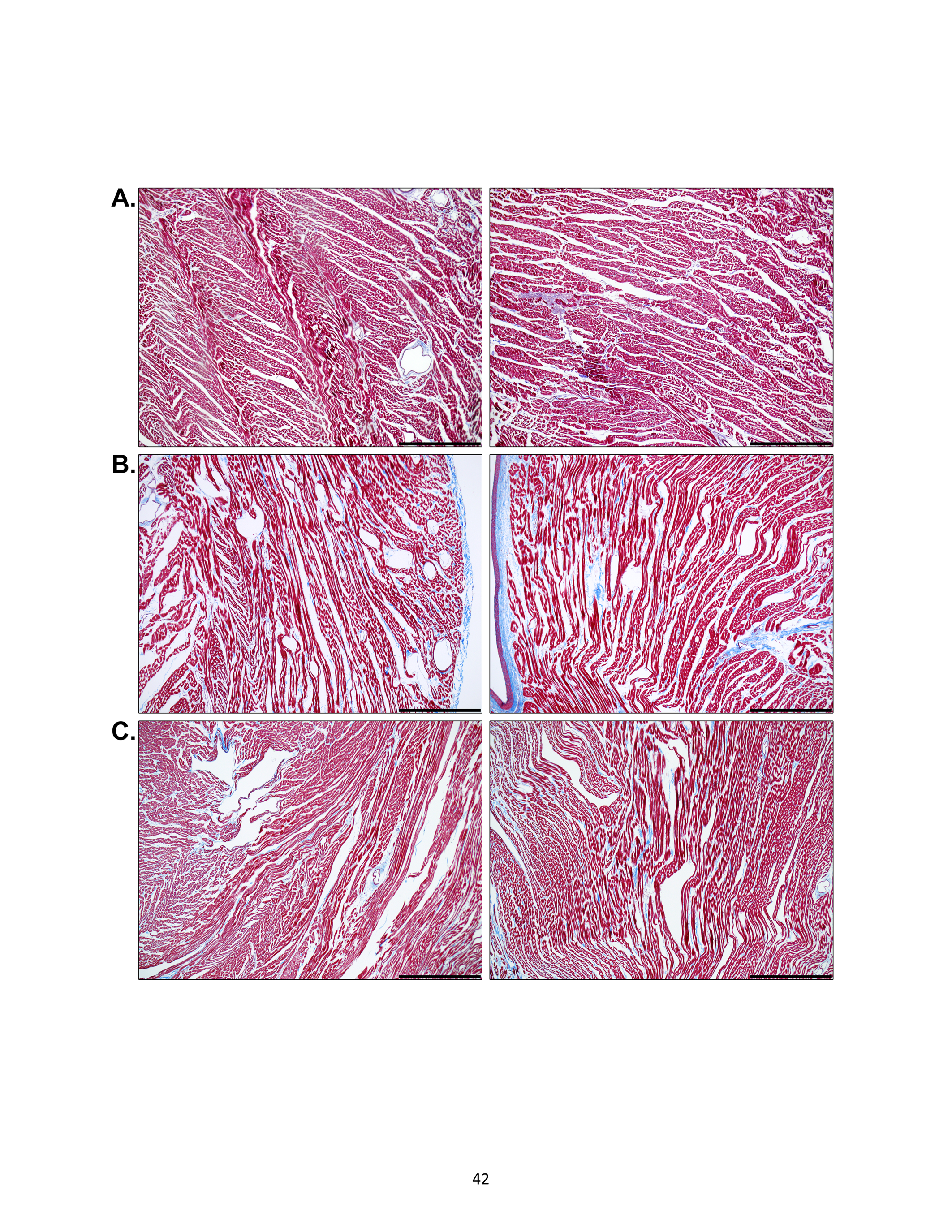

Supplement: S4 Fig — (A) Sham, (B) Control, and (C) Mitochondria experimental groups are shown. Non-ischemic (A) and regionally ischemic (B and C) rabbit hearts were injected (left panels) or perfused (right panels) with vehicle (B) or 1 x 10^8^ mitochondria (C). Scale bars equal 500 μm. (TIF) [file pone.0160889.s004.tif]
